# Localized Angiosarcoma, Not One Disease: A Retrospective Single-Center Study on Prognosis Depending on the Primary Site and Etiology

**DOI:** 10.1155/2021/9960085

**Published:** 2021-09-10

**Authors:** Inna Schott, Sven-Thorsten Liffers, Farhad Farzaliyev, Johanna Falkenhorst, Hans-Ulrich Steinau, Jürgen-Walter Treckmann, Lars Erik Podleska, Christoph Pöttgen, Hans-Ulrich Schildhaus, Marit Ahrens, Uta Dirksen, Fatma-Zehra Murat, Jens T. Siveke, Sebastian Bauer, Rainer Hamacher

**Affiliations:** ^1^Department of Medical Oncology, Sarcoma Center, West German Cancer Center, University Hospital Essen, Essen, Germany; ^2^Bridge Institute of Experimental Tumor Therapy, West German Cancer Center, University Hospital Essen, Essen, Germany; ^3^Division of Solid Tumor Translational Oncology, German Cancer Consortium (DKTK,Partner Site Essen) and German Cancer Research Center, DKFZ, Heidelberg, Germany; ^4^Department of Orthopedic Oncology, West German Cancer Center, University Hospital Essen, Essen, Germany; ^5^German Cancer Consortium (DKTK), Partner Site University Hospital Essen, Essen, Germany; ^6^Department of General, Visceral and Transplantion Surgery, Sarcoma Center, West German Cancer Center, University Hospital Essen, Essen, Germany; ^7^Department of Radiotherapy, West German Cancer Center, University Hospital Essen, Essen, Germany; ^8^Institute of Pathology, West German Cancer Center, University Hospital Essen, Essen, Germany; ^9^Medical Clinic II, University Hospital Frankfurt, Frankfurt am Main, Germany; ^10^Pediatrics III Pediatric Hematology, Oncology, Immunology, Cardiology, Pulmonology, West German Cancer Center, University Hospital Essen, Essen, Germany

## Abstract

**Background:**

Angiosarcomas are rare and heterogeneous tumors with poor prognosis. The clinical subtypes are classified depending on the primary site and etiology.

**Methods:**

We conducted a retrospective, monocentric study of 136 patients with localized AS between May 1985 and November 2018. Overall survival (OS), local recurrence-free survival (LRFS), and metastasis-free survival (MFS) were estimated using the Kaplan–Meier method. To identify prognostic factors, univariate and multivariate analyses were performed based on Cox regressions.

**Results:**

The median age was 67 years (19–72.8 years). Primary sites were cutaneous (27.2%), breast (38.2%), and deep soft tissue (34.6%). The majority was primary angiosarcomas (55.9%) followed by postradiation (40.4%) and chronic lymphedema angiosarcomas (2.9%). Prognosis significantly differed depending on the primary site and etiology. Shortest median OS and MFS were observed in deep soft tissue angiosarcomas, whereas cutaneous angiosarcomas, angiosarcomas of the breast, and radiation-associated angiosarcomas displayed worse median LRFS. Univariate analyses showed better OS for tumor size <10 cm (*p* = 0.009), negative surgical margins (*p* = 0.021), and negative lymph node status (*p* = 0.007). LRFS and MFS were longer for tumor size <10 cm (*p* = 0.012 and *p* = 0.013). In multivariate analyses, age <70 years was the only independent positive prognostic factor for OS in all subgroups. For LRFS, secondary AS of the breast was a negative prognostic factor (HR: 2.35; *p* = 0.035).

**Conclusions:**

Different behaviors and prognoses depending on the primary site and etiology should be considered for the treatment of this heterogeneous disease. In cutaneous angiosarcomas of the head/neck and postradiation angiosarcomas of the breast, local recurrence seems to have a crucial impact on OS. Therefore, improved local therapies and local tumor staging may have to be implemented. However, in deep soft tissue angiosarcomas, distant recurrence seems to have a major influence on prognosis, which indicates a benefit of additional perioperative chemotherapy.

## 1. Introduction

Angiosarcomas are very rare and malignant tumors that share markers of differentiation with endothelial cells of blood or lymphatic vessels [1]. These tumors account for only 2–5% of soft tissue sarcomas with an incidence of only 1.5 to 2.8 per million per year [1–5]. Angiosarcomas occur everywhere in the body as primary tumors or secondary tumors related to radiation therapy or chronic lymphedema. Depending on the anatomic location and etiology, they are typically subdivided into cutaneous angiosarcomas, mainly on the head and neck, radiation-induced angiosarcomas, mainly of the breast, lymphedema-associated angiosarcomas, primary breast angiosarcomas, and soft tissue angiosarcomas [1]. The prognosis of angiosarcomas is generally poor with median overall survival reported between 30 and 50 months and 5-year overall survival rates between 10% and 50%, while localized angiosarcomas range around 40%, and metastasized disease drops to around 15% [1, 5–8]. The impact of the type of angiosarcoma on prognosis is not well studied, and particularly, generic staging classifications appear to have limited value in angiosarcomas. However, an increasing number of studies demonstrate distinct biological and clinical differences between certain subtypes. Cutaneous angiosarcomas of the head and neck have been described to be associated with high tumor mutational burden (TMB) and dominant ultraviolet damage mutational signature which makes them particular candidates for immune checkpoint therapy [9–11]. Radiation- and lymphedema-associated angiosarcomas were associated with MYC gene amplification, whereas primary angiosarcomas of the breast showed a high rate of PIK3CA-activating mutations [11, 12]. Regarding the treatment of localized angiosarcomas, randomized clinical trials do not exist. Few studies investigated the relevance of perioperative treatment with radiation therapy and chemotherapy. However, general recommendation cannot not be drawn yet [1, 13–16]. In this context, angiosarcomas are treated according to other high-grade sarcomas with the primary aim of complete resection with wide margins [1].

In this retrospective single-center study, we systematically addressed the value of several factors in localized angiosarcomas with focus on the main clinical subtypes according to the anatomic site and etiology. Our study demonstrates a distinct different behavior of the subtypes with regard to local recurrence, metastasis, and overall survival. While deep soft tissue angiosarcomas showed a high risk of metastasis, in cutaneous angiosarcomas, angiosarcomas of the breast, and secondary angiosarcomas, the risk of local recurrence was increased. Risk factors for reduced survival were tumor size >10 cm, positive surgical margins and positive or unknown lymph node status, and age >70 years.

## 2. Materials and Methods

### 2.1. Study Design and Population

Patients with the diagnosis of angiosarcoma between May 1st, 1985, and November 30th, 2018, were identified using the institutional tumor documentation database of the West German Cancer Center, University Hospital Essen, Essen, Germany. All consecutive patients with a pathological diagnosis of angiosarcoma were entered in the electronic database. Angiosarcoma was defined using the International Classification of Diseases for Oncology, 3rd edition (ICD-O-3), morphological code 9120/3. Age at diagnosis, sex, histology, etiology, tumor size, tumor location, nodal status, occurrence and date of local relapse, occurrence, location, and date of metastasis, surgery (extent of resection: R0, R1, or R2), perioperative treatment, and date of death/last encounter were collected for each patient by the retrospective chart review. Angiosarcoma patients were classified based on etiology (spontaneous, radiation-, and lymphedema-associated) and anatomic location (cutaneous, breast/trunk, and deep tissue including patients with angiosarcoma of organs). Time intervals for the analysis were calculated from the date of diagnosis.

### 2.2. Statistical Considerations

Descriptive statistical analysis was used. Therefore, frequency of occurrence and percentage were calculated for each of the independent variables. Overall survival (OS), local recurrence-free survival (LRFS), metastasis-free survival (MFS), and survival rates (2- and 5-year) were calculated using the Kaplan–Meier method and survival tables. Differences in OS, LRFS, and MFS were determined using the log-rank test. To identify prognostic factors for survival, univariable analyses were performed using Cox regression analysis. On the basis of clinical relevance and significance in univariable tests, factors were selected for multivariate Cox regression analysis. Statistical analyses were performed with IBM SPSS Statistics version 27 (Armonk, NY, USA) and the R survival package (Therneau T (2020); a package for survival analysis in R; version 3.2.7, http://www.r-project.org) using R (version 3.6.3, http://www.r-project.org).

## 3. Results

### 3.1. Demographics and Disease Characteristics

The demographic, tumor, and treatment characteristics are presented in Table 1. We identified 136 patients with localized disease (UICC II-IIIB) at the time of diagnosis with a slight dominance of female patients. The majority of patients was younger than 70 years at diagnosis. Most patients presented with spontaneous angiosarcomas, followed by radiation-associated angiosarcomas and only four with lymphedema-associated angiosarcoma, so called Stewart–Treves syndrome. The primary location was the breast in 52 patients (38.2%) and other deep tissues in 47 patients (34.6%), and 37 patients were with cutaneous angiosarcoma (27.2%). For the majority of patients, the tumor size was ≤5 cm. The distribution of tumors >5 cm and ≥10 cm was similar. However, in a significant number of patients, the tumor size was not known. For 119 patients, comprehensive data on treatment were available, where in 69 patients (58.0%), only surgery was performed and 50 patients (42%) received perioperative treatment. Most patients presented with a negative lymph node status, and R0 resection was achieved in 47.8%. However, in a significant number of patients, the initial lymph node status and margin status could not be determined. Metachronous metastasis was observed in 45 patients (33.1%).

### 3.2. Prognostic Factors for Overall Survival

**Figure 1:**
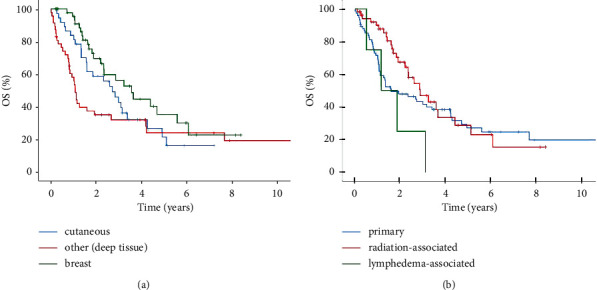
Overall survival (OS). Kaplan–Meier curves showing 10-year OS (a) comparing deep soft tissue angiosarcomas (*N* = 47), cutaneous angiosarcomas (*N* = 37), and angiosarcomas of the breast (*N* = 52) with a median OS of 12.7 vs. 35.6 (*p* = 0.213) and 42.6 months (*p* = 0.006^*∗∗*^) and (b) comparing primary (*N* = 76), radiation-associated (*N* = 55), and lymphedema-associated (*N* = 4) angiosarcomas with a median OS of 18.9, 34.2, and 13.6 months (*p* > 0.05).

The 2-year and 5-year overall survival rates for the entire cohort of localized angiosarcoma were 54% and 30%, respectively. The median overall survival was 31.8 months. Kaplan–Meier curves showed a significant reduced median overall survival for deep soft tissue angiosarcomas compared to cutaneous angiosarcomas and angiosarcomas of the breast (Figure 1(a)). Hereby, angiosarcomas of the breast, which were mainly radiation-associated, also showed a better 5-year survival rate with 38% compared to cutaneous angiosarcomas with 21% and deep soft tissue angiosarcomas with 25% (Figure 1(a)). A slightly better outcome was demonstrated in radiation-associated versus spontaneous angiosarcomas, although not statistically significant (Figure 1(b)). The four patients with lymphedema-associated angiosarcoma showed the shortest median overall survival, and all died during follow-up time (Figure 1(b)). Univariate analyses for parameters of tumor staging showed a significantly reduced median overall survival for tumor size ≥10 cm, positive or unknown lymph mode status, and positive surgical margins (Table 2).

### 3.3. Prognostic Factors for Local Recurrence-Free Survival

**Figure 2:**
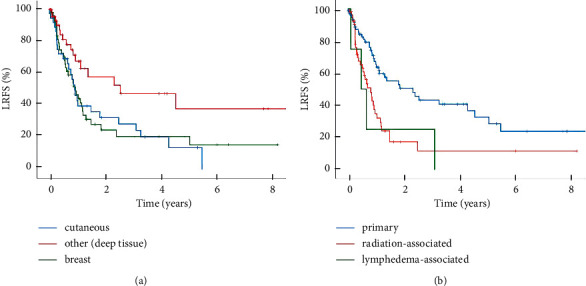
Local recurrence-free survival (LRFS). Kaplan–Meier curves showing 8-year LRFS (a) comparing deep soft tissue angiosarcomas (*N* = 47), cutaneous angiosarcomas (*N* = 37), and angiosarcomas of the breast (*N* = 52) with a median LRFS of 30.2 vs. 10.7 vs. 11.0 months (*p* = 0.013^∗^; *p* = 0.018^∗^) and (b) comparing primary (*N* = 77), radiation-associated (*N* = 45), and lymphedema-associated (*N* = 4) angiosarcomas with a median LRFS of 27.6 vs. 9.6 vs. 5.5 months (*p* = 0.001^∗∗∗^; *p* = n.a.).

Next, we analyzed the factors that determine the risk of local relapse. The entire cohort of localized angiosarcomas showed a 2-year and 5-year local recurrence-free rates of 22% and 14%, respectively. The median local recurrence-free survival was 12.2 months. For deep soft tissue angiosarcomas, the median local recurrence-free survival was significantly longer compared to cutaneous angiosarcomas and angiosarcomas of the breast (Figure 2(a)). Regarding the etiology, primary angiosarcomas showed significantly longer local recurrence-free survival compared to radiation-associated angiosarcomas (Figure 2(b)). The four patients with lymphedema-associated angiosarcoma had shortest local recurrence-free survival (Figure 2(b)). Additionally, univariate analysis revealed that tumor size >10 cm was associated with a significantly reduced local recurrence-free survival in the entire cohort of localized angiosarcomas (Table 2). Due to the small number, the subentities were excluded for further analysis. Moreover, patients ≥70 years showed a reduced local recurrence-free survival (Table 2).

### 3.4. Prognostic Factors for Metastasis-Free Survival

**Figure 3:**
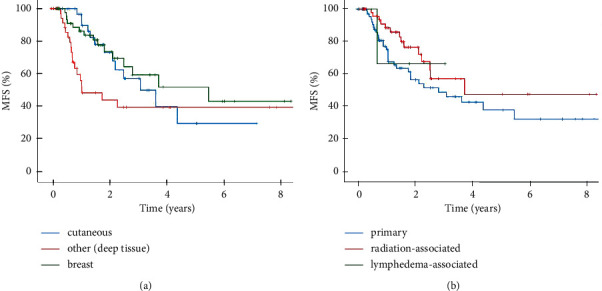
Metastasis-free survival (MFS). Kaplan–Meier curves showing 8-years MFS (a) comparing deep soft tissue angiosarcomas (*N* = 46), cutaneous angiosarcomas (*N* = 37), and angiosarcomas of the breast (*N* = 52) with a median MFS of 13.0 vs. 44.0 vs. 66.0 months (*p* = 0.068; *p* = 0.034^*∗*^) and (b) comparing primary (*N* = 77) and radiation-associated (*N* = 55) angiosarcomas with a median MFS of 34.2 vs. 45.1 (*p* = 0.103).

The median metastasis-free survival for the cohort of localized angiosarcomas was 44.0 months. Similar to the analysis for overall survival, deep soft tissue angiosarcomas showed a reduced median metastasis-free survival compared to cutaneous angiosarcomas and angiosarcomas of the breast, at which was significant to the latter (Figure 3(a)). The difference in metastasis-free survival between primary and radiation-associated angiosarcomas was not significant (Figure 3(b)). Only one patient with lymphedema-associated angiosarcoma showed distant metastases to the thigh and femoral head at 8.4 months after diagnosis. Similar to the previous analysis, tumor size >10 cm is associated with a significantly lower median metastasis-free survival (Table 2).

### 3.5. Multivariate Analyses

**Figure 4:**
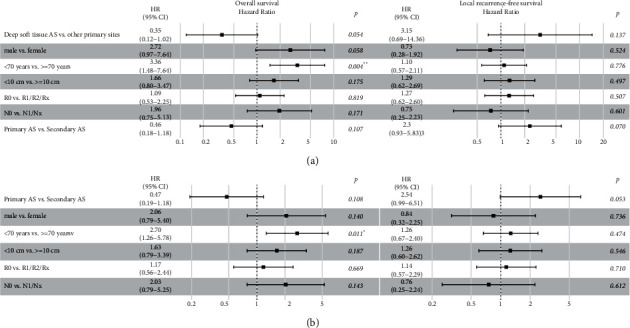
Multivariate analysis. Forest plots of multivariate Cox regression analysis on overall survival (left) and local recurrence-free survival (right) for the subgroups (a) deep soft tissue angiosarcomas and (b) primary vs. secondary angiosarcomas showing hazard ratio (HR), 95% confidence interval (CI), and *p* value.

To identify independent prognostic factors, we performed multivariate Cox regression analyses on overall, local recurrence-free, and metastasis-free survival. The only significant prognostic factor for better overall survival in all subgroups was age <70 years (Figure 4 and Supplement Figure 1). The primary site had no significant impact on survival in multivariate analyses. However, similar to Kaplan–Meier analyses, prognosis for overall survival was in disfavor of deep soft tissue angiosarcomas (Figure 4(a)). For local recurrence-free survival, the etiology ‘secondary angiosarcoma' was identified as a negative prognostic factor in all subgroups, but was only significant for angiosarcomas of the breast (Supplement Figure 1). For metastasis-free survival, no significant prognostic factor by multivariate Cox regression analysis could be detected (Supplement Figure 2).

## 4. Discussion

In this retrospective single-center study, we evaluated the prognostic value of clinical factors with regard to overall survival as well as local recurrence-free and metastasis-free survival in patients with localized angiosarcoma. The rarity and heterogeneity of angiosarcomas pose barriers to comprehensive studies.

Our study is one of the largest single-center studies for localized angiosarcomas with 136 patients, compared to 28–324 patients in other studies, whereas not all focused on localized disease [5, 6, 8, 17–21]. The median age in our cohort with 67 years and a slight dominance of female patients are comparable to other cohorts [5, 6, 8, 17–21]. In contrast to other studies, we systematically focused on localized angiosarcomas and compared the main clinical subtypes, namely, cutaneous angiosarcomas, angiosarcomas of the breast, deep soft tissue angiosarcomas, and primary and radiation-associated angiosarcomas. We also included lymphedema-associated angiosarcomas in our analysis, but with only four patients, no statistical analyses could be performed. Overall, the distribution of angiosarcoma subtypes is comparable to other studies, although a high variability can be observed between studies: cutaneous angiosarcomas (13–75%), angiosarcomas of the breast (16–56%), deep soft tissue angiosarcomas (5–42%), and primary (46–71%), radiation-associated (17–63%), and lymphedema-associated (3–22%) angiosarcomas [5, 6, 8, 17–21]. The overall survival for localized angiosarcomas varies in other studies with a median time from 20.8 to >60 months and 5-year survival rates from 17.9% to 74.8%, whereas our cohort ranges somehow in the middle [5, 6, 8, 17–19, 21]. Local recurrence-free survival and metastasis-free survival were, in most studies, not systematically investigated. The 2-year local control rates are reported with 58% to 75%, which are a bit higher compared to our cohort [6, 8, 17, 20]. This difference might be explained by the fact that many patients in our cohort were not primarily resected at a specialized center and only referred subsequently to our sarcoma center. Median metastasis-free survival was reported lower in one study with 36.1 months compared to our cohort, and 2-year distant control rates were reported from 46.4% to 81.4% [6, 8, 20]. This high variation is very likely due to the high heterogeneity of clinical subtypes summarized under the umbrella of angiosarcomas. Therefore, our study aimed to compare the subtypes of angiosarcoma and to evaluate prognostic factors.

While other studies reported a shorter median overall survival for cutaneous angiosarcomas (19.7–29 months), the 2- and 5-year overall survival rates were comparable [22–25]. Here, the site of the cutaneous angiosarcomas seems important, with a worse prognosis for angiosarcomas of the scalp compared to the face/neck and outside the head/neck [19, 22, 25]. In our cohort, the majority was located at the head and neck, and due to our reporting system, discrimination between the scalp, face, and neck was not possible. Regarding local recurrence in cutaneous angiosarcomas, most studies reported a better 5-year local control rate between 18% and 43% [22, 23]. Metastasis-free survival was not systematically investigated in other studies [22–25]. For angiosarcomas of the breast, two meta-analyses with patient numbers of 222 [26] and 975 [27] and one retrospective study (*N* = 49) [28] presented comparable data for overall survival, with a 2-year overall survival rate of 71.1% [28], a 5-year overall survival rate of 43% [26], and a wide range for median overall survival from 12 to 72 months, with a comparable average to our cohort of around 43 months [27]. Other retrospective studies observed higher 5-year rates for overall survival (61–69%) [29, 30]. The local control in other studies on angiosarcomas of the breast showed a better prognosis for 2 years with 55.2%, 5 years from 44% to 62%, and median recurrence-free survival from 6 to 54 months, averaging at 18 months [26–29]. Our cohort contained mainly radiation-associated angiosarcomas, which might explain the high risk of local recurrence. This was supported by the multivariate analyses, where secondary angiosarcoma was the only significant risk factor for local recurrence. Only limited data are published on metastasis, which is generally seen with low risk for angiosarcomas of the breast, with a reported 2-year metastasis-free survival rate of 57.3% and median time of 13.2 months [28, 29]. Regarding deep soft tissue angiosarcomas, to our knowledge, no study so far focused on the analysis of localized disease. However, these deep soft tissue angiosarcomas were included in studies about the prognosis of angiosarcomas in general or studies focused on certain organs, such as the liver, kidney, lung, or heart [1, 5, 6, 8, 17]. Here, median overall survival was reported similarly poor between 2.8 and 18.2 months [5, 6, 8, 21]. One study also addressed local recurrence and metastasis, showing a lower local recurrence-free survival of 17 months and comparable poor metastasis-free survival of 11 months [8].

Our study demonstrates a similar behavior of cutaneous angiosarcomas and angiosarcomas of the breast with comparable median overall survival, local recurrence-free survival, and metastasis-free survival. Notably, the median metastasis-free survival was longer than the median overall survival, suggesting that a significant number of patients died of their local relapse. This is underscored by a very short median local recurrence-free survival for both subtypes, which is in accordance with other studies [8, 24, 26]. On the contrary, deep soft tissue angiosarcomas showed worse median overall survival, which was associated with reduced median metastasis-free survival time.

In our cohort, we also compared primary to secondary angiosarcomas, namely, radiation- and lymphedema-associated. In contrast to several other studies, we did not separate UV-associated angiosarcomas from primary angiosarcomas. The worst prognosis was seen for the four lymphedema-associated angiosarcomas, which is in accordance with previous reports [5, 8]. For radiation-associated angiosarcomas, we observed a nonsignificant trend towards a better median overall survival and metastasis-free survival compared to primary angiosarcomas. The opposite was seen for local recurrence-free survival, which was significantly worse. Here, a clear tendency was also observed in the multivariate analysis, but only significant for secondary angiosarcomas of the breast. The results from other studies are diverse. While some do not observe a significant difference in overall survival [18, 31, 32], other studies showed better or worse prognosis for primary vs. secondary angiosarcomas [5, 20]. Two studies confirmed the worse prognosis of radiation-associated angiosarcomas in local recurrence (mLRFS: 20.1 months vs. 38 months; 2 y LC: 31.9% vs. 72.4%) but showed contradicting results with worse prognosis as well in overall survival (mOS: 26.5 months vs. 39.9–45.9 months; 2 y OS: 45% vs. 65%) and distant free survival (mDFS: 26.1 months vs. 29–39 months; 2 y DC: 41.4% vs. 73.4%) [8, 20].

To obtain prognostic factors, we performed univariate and multivariate analyses. We excluded tumor grade as a covariate as this is no longer considered applicable to angiosarcomas [33]. In univariate analyses, tumor size >10 cm, positive surgical margin, and positive or unknown lymph node status were significantly associated with worse overall survival in the cohort of all localized angiosarcomas. Hereby, tumor size >10 cm was also significantly associated with shorter local recurrence-free and metastasis-free survival, but multivariate analyses did not confirm tumor size, surgical margin, and lymph node status as independent prognostic factors. This might be due to the high number of unknown tumor sizes and not evaluable surgical margins and lymph node status. With regard to size, estimation of tumor extent could be vastly underestimated in patients with any skin-associated angiosarcoma. For these patients, surgical margins may also be more difficult to determine as tumor cells grow rather diffuse and therefore leave more frequently cutaneous skip metastases. Previous studies have reported tumor size, mostly >5 cm [6, 8, 24, 26–28, 30], and positive surgical margins as negative prognostic factors in angiosarcomas [8, 21, 23]. In line with previous studies, the only independent positive prognosis factor for overall survival, observed in all clinical subtypes, was age <70 years [5, 8, 19, 24, 26].

Our study demonstrates a distinct different behavior of the subtypes with regard to local recurrence, metastasis, and overall survival. While deep soft tissue angiosarcomas showed a high risk of metastasis, in cutaneous angiosarcomas, angiosarcomas of the breast, and secondary angiosarcomas, the risk of local recurrence was increased. There are several limitations. Although the number of localized angiosarcomas is high and comparable to other studies, the numbers in the subgroups are too low to perform valid statistical analysis for all prognostic factors within the subgroups. Moreover, the clinical subgroups of cutaneous angiosarcomas, angiosarcomas of the breast, and deep soft tissue angiosarcomas are still quite heterogeneous. We did not consider the exact anatomic site or distinguish the etiology within each of the subgroups (cutaneous angiosarcoma, angiosarcoma of the breast, and deep soft tissue angiosarcoma). Moreover, for many patients, clinical information on tumor size, surgical margins, and lymph node status was, due to the retrospective analysis, not determinable.

Nevertheless, our study provides several implications for consulting patients, therapy planning, and future clinical studies. Our data indicate a high risk of local recurrence and lower risk for early metastasis in cutaneous angiosarcomas and angiosarcomas of the breast, whereas for the latter, our data are predominantly based on secondary angiosarcomas. These are rather angiosarcomas of the chest wall and behave clinically different to primary angiosarcomas of the breast. As discussed above, local tumor control may have a major impact on survival. However, this is hampered by the fact that cutaneous and radiation- and lymphedema-associated angiosarcomas are not contained within a compartment, but often show blurry margins, and neither inspection nor imaging allows to exactly determine the extent of the disease. Due to this, the tumor size often cannot be determined, and TNM classification for the primary site seems obsolete. Here, improved methods for local staging have to be investigated and implemented. In addition to the difficulty to determine the exact tumor expansion, often, limited perioperative therapy options are available, especially in radiation-associated angiosarcomas, due to pretreatment for initial malignant disease. Radical surgical resection represents, by far, the most important therapeutic modality to cure these tumors. For radiation-associated angiosarcomas of the chest wall, radical excision that includes the complete irradiated field followed by chest wall reconstruction had a significant positive impact on local control, distant metastasis, and disease-specific survival [34, 35]. Such radical resection is technically impossible in many patients with cutaneous angiosarcomas of the head and neck and lymphedema-associated angiosarcomas. The role of multimodal approaches, such as hyperthermic isolated limb perfusion with TNF-alpha and melphalan [36] or perioperative chemo- and radiotherapy, is yet disputed, but many centers consider it as part of primary treatment in younger patients with good performance status [8, 14, 15, 27, 36]. For deep soft tissue angiosarcomas, distant disease control seems to be particularly important. Recently, Pasquali et al. demonstrated a better survival for patients with low predicted overall survival when adjuvant chemotherapy was applied, which is true for most angiosarcomas [37]. Unfortunately, retrospective analyses from randomized trials do not allow conclusions due to the low numbers of angiosarcoma patients that were included. Another topic is recommendations for follow-up after curative treatment which are not standardized, and evidence-based data are missing. Interestingly, the Kaplan–Meier plots for overall survival, local recurrence-free survival, and metastasis-free survival in our cohort show a plateau between 5 and 6 years for all clinical subtypes, which is similar to other studies [5, 6, 8, 20, 21]. This suggests that follow-up in angiosarcomas should be planned for a minimum of 5 to rather 6 years.

## 5. Conclusion

Angiosarcomas represent a heterogeneous sarcoma subtype with strikingly different clinical behaviors and prognoses depending on the primary site and etiology. Local control should be the focus for cutaneous angiosarcomas, especially of the head/neck, and radiation-associated angiosarcomas as local relapses define the outcome for most patients. Therapeutic concepts should incorporate early and aggressive surgical and, wherever possible, radiotherapeutic treatment. Given the very high risk of the metastatic disease in deep soft tissue angiosarcomas, long-term survival will only improve with the use of systemic treatments. The lack of prospective and randomized trials for this subgroup of patients poses a considerable challenge for advising patients.

## Figures and Tables

**Table 1:** Patient characteristics for the cohort of localized angiosarcomas.

		*N*	%
Follow-up time	Median follow-up time: 19.1 months (0.1–428.0 months)	136	

Age	Median age: 67 years (19–72.8 years)	136	
	<70 years	78/136	57.4
	≥70 years	58/136	42.6

Sex	Female	81/136	59.6
	Male	55/136	40.4

Tumor size	≤5 cm	50/136	36.8
	>5 cm	19/136	14.0
	≥10 cm	21/136	16.2
	Unknown	45/136	33.1

Etiology	Primary	76/136	55.9
	Postradiation	55/136	40.4
	Chronic lymphedema	4/136	2.9
	Unknown	1/136	0.7

Localization	Cutaneous (i) Head and neck (ii) Others	37/136 25/136 12/136	27.2 18.4 8.8
	Breast	52/136	38.2
	Others (deep tissue) (i) Abdomen (ii) Thorax (iii) Limbs (iv) Others	47/136 15/136 9/136 16/136 7/136	34.6 11.0 6.6 11.8 5.1

Lymph node	N0	87/136	64.0
	N1	8/136	5.9
	Nx	41/136	30.1

Margin status	R0	65/136	47.8
	R1	26/136	19.1
	R2	4/136	2.9
	Rx	23/136	16.9
	No surgery	18/136	13.2

Treatment	Only surgery	69/119	58.0
	Perioperative therapy	50/119	42.0

Metachronous metastasis	All	45/136	33.1
	Cutaneous	12/136	8.8
	Breast	15/136	11.0
	Others (deep tissue)	19/136	14.0

**Table 2:** Univariate analysis for median overall survival, local recurrence-free survival, and metastasis-free survival displayed in months and *p* value.

Categories		Prognostic factors for					
		Overall survival		Local recurrence-free survival		Metastasis-free survival	
		Median OS (months)	*p*	Median LRFS (months)	*p*	Median MFS (months)	*p*
Gender	Female	33.9		11.0		45.1	
	Male	27.7	0.5	21.5	0.055	37.5	0.64

Age	<70 years	28.0		16.4		34.2	
	≥70 years	31.0	0.24	11.0	0.045^*∗*^	45.1	0.225

Tumor size
	≤5 cm (*N* = 49)	52.7		21.5		44.0	
	>5 cm (*N* = 19)	56.1	0.94	15.6	0.58	43.5	0.837
	>10 cm (*N* = 13)	19.9	0.009^*∗∗*^	8.0	0.012^*∗*^	17.0	0.013^*∗*^
	>15 cm (*N* = 9)	18.9	0.559	6.0	0.612	18.9	0.38

Lymph node	N0	34.2		12.0		13.0	
	N1/Nx	18.9	0.007^*∗∗*^	7.7	0.587	8.7	0.971

Margins	R0	38.5		12.2		53.0	
	R1, R2	15.9	0.021^*∗*^	9.0	0.74	22.4	0.077

^*∗*^*p* ≤ 0.05; ^*∗∗*^*p* ≤ 0.01; ^*∗∗∗*^*p* ≤ 0.001.

## Data Availability

The datasets used and/or analyzed during the present study are available from the corresponding author upon reasonable request.

## Abbreviations

AS: Angiosarcoma
CI: Confidence interval
cm: Centimeters
DC: Distant control
mDFS: Median distant free survival
HR: Hazard ratio
LC: Local control
LRFS: Local recurrence-free survival
MFS: Metastasis-free survival
mLRFS: Median local recurrence-free survival
mMFS: Median metastasis-free survival
mOS: Median overall survival
*N*: Number
n.a.: Not applicable
OS: Overall survival
OP: Operation
*p*: *p* value
PF: Prognostic factor
Tx: Therapy
UV: Ultraviolet
vs.: Versus
y: Years.

## Supplementary Materials

Supplementary Materials Supplement Figure 1: multivariate analysis. Forest plots of multivariate Cox regression analysis on overall survival (left) and local recurrence-free survival (right) for the subgroups A) cutaneous angiosarcomas and B) angiosarcomas of the breast showing hazard ratio (HR), 95% confidence interval (CI), and *p* value. Supplement Figure 2: multivariate analysis of metastasis-free survival. Forest plots of multivariate Cox regression analysis on metastasis-free survival for the subgroups A) cutaneous angiosarcomas, B) angiosarcomas of the breast, C) deep soft tissue angiosarcomas, and D) primary vs. secondary angiosarcomas showing hazard ratio (HR), 95% confidence interval (CI), and *p* value.

## References

[1] Young R. J., Brown N. J., Reed M. W., Hughes D., Woll P. J. (2010). Angiosarcoma. *The Lancet Oncology*.

[2] De Pinieux G., Karanian M., Le Loarer F. (2021). Nationwide incidence of sarcomas and connective tissue tumors of intermediate malignancy over four years using an expert pathology review network. *PLoS One*.

[3] Dennis NF M., Lawrence G. (2012). Soft tissue sarcoma incidence and survival tumours diagnosed in england between 1985 and 2009. http://wwwncinorguk/cancer_type_and_topic_specific%20work/cancer_type_specific_work/sarcomas/.

[4] Trama A., Badalamenti G., Baldi G. G. (2019). Soft tissue sarcoma in Italy: from epidemiological data to clinical networking to improve patient care and outcomes. *Cancer Epidemiology*.

[5] Weidema M. E., Flucke U. E., Van der Graaf W. T. A. (2019). Prognostic factors in a large nationwide cohort of histologically confirmed primary and secondary angiosarcomas. *Cancers*.

[6] Buehler D., Rice S. R., Moody J. S. (2014). Angiosarcoma outcomes and prognostic factors. *American Journal of Clinical Oncology*.

[7] Florou V., Wilky B. A. (2018). Current and future directions for angiosarcoma therapy. *Current Treatment Options in Oncology*.

[8] Lindet C., Neuville A., Penel N. (2013). Localised angiosarcomas: the identification of prognostic factors and analysis of treatment impact. A retrospective analysis from the French Sarcoma Group (GSF/GETO). *European Journal of Cancer*.

[9] Florou V., Rosenberg A. E., Wieder E. (2019). Angiosarcoma patients treated with immune checkpoint inhibitors: a case series of seven patients from a single institution. *Journal for ImmunoTherapy of Cancer*.

[10] Hamacher R., Kämpfe D., Reuter-Jessen K. (2018). Dramatic response of a PD-L1-positive advanced angiosarcoma of the scalp to pembrolizumab. *JCO Precision Oncology*.

[11] Painter C. A., Jain E., Tomson B. N. (2020). The Angiosarcoma Project: enabling genomic and clinical discoveries in a rare cancer through patient-partnered research. *Nature Medicine*.

[12] Udager A. M., Ishikawa M. K., Lucas D. R., McHugh J. B., Patel R. M. (2016). MYC immunohistochemistry in angiosarcoma and atypical vascular lesions: practical considerations based on a single institutional experience. *Pathology*.

[13] Esposito E., Avino F., di Giacomo R. (2019). Angiosarcoma of the breast, the unknown-a review of the current literature. *Translational Cancer Research*.

[14] Lee K. T., Moon J., Jeong H. S., Lim H. S., Lim S. Y. (2020). Benefits of the multidisciplinary approach after curative surgery for the treatment of scalp angiosarcoma. *Annals of Plastic Surgery*.

[15] Oxenberg J., Khushalani N. I., Salerno K. E., Attwood K., Kane J. M. (2015). Neoadjuvant chemotherapy for primary cutaneous/soft tissue angiosarcoma: determining tumor behavior prior to surgical resection. *Journal of Surgical Oncology*.

[16] Rombouts A. J. M., Huising J., Hugen N. (2019). Assessment of radiotherapy-associated angiosarcoma after breast cancer treatment in a Dutch population-based study. *JAMA Oncology*.

[17] Fayette J., Martin E., Piperno-Neumann S. (2007). Angiosarcomas, a heterogeneous group of sarcomas with specific behavior depending on primary site: a retrospective study of 161 cases. *Annals of Oncology*.

[18] Lahat G., Dhuka A. R., Hallevi H. (2010). Angiosarcoma. *Annals of Surgery*.

[19] Lee B. L., Chen C. F., Chen P. C. (2017). Investigation of prognostic features in primary cutaneous and soft tissue angiosarcoma after surgical resection: a retrospective study. *Annals of Plastic Surgery*.

[20] Merfeld E., Gabani P., Spraker M. B. (2019). Clinical outcomes and prognostic features of angiosarcoma: significance of prior radiation therapy. *Clinical Oncology*.

[21] Smrke A., Hamm J., Karvat A., Simmons C., Srikanthan A. (2020). A retrospective review of 145 patients with angiosarcoma: radiation therapy, extent of resection and chemotherapy are important predictors of survival. *Molecular and Clinical Oncology*.

[22] Bernstein J. M., Irish J. C., Brown D. H. (2017). Survival outcomes for cutaneous angiosarcoma of the scalp versus face. *Head & Neck*.

[23] Kuan C. H., Yang H. W., Huang H. F. (2020). Prognostic significance of positive surgical margins for scalp angiosarcoma. *Journal of the Formosan Medical Association*.

[24] Lee K. C., Chuang S.-K., Philipone E. M., Peters S. M. (2019). Characteristics and prognosis of primary head and neck angiosarcomas: a surveillance, epidemiology, and end results program (seer) analysis of 1250 cases. *Head and Neck Pathology*.

[25] Moon I. J., Kim Y. J., Won C. H. (2020). Clinicopathological and survival analyses of primary cutaneous angiosarcoma in an Asian population: prognostic value of the clinical features of skin lesions. *International Journal of Dermatology*.

[26] Depla A. L., Scharloo-Karels C. H., de Jong M. A. A. (2014). Treatment and prognostic factors of radiation-associated angiosarcoma (RAAS) after primary breast cancer: a systematic review. *European Journal of Cancer*.

[27] Abdou Y., Elkhanany A., Attwood K., Ji W., Takabe K., Opyrchal M. (2019). Primary and secondary breast angiosarcoma: single center report and a meta-analysis. *Breast Cancer Research and Treatment*.

[28] Cohen-Hallaleh R. B., Smith H. G., Smith R. C. (2017). Radiation induced angiosarcoma of the breast: outcomes from a retrospective case series. *Clinical Sarcoma Research*.

[29] Salminen S. H., Wiklund T., Sampo M. M. (2020). Treatment and prognosis of radiation-associated breast angiosarcoma in a nationwide population. *Annals of Surgical Oncology*.

[30] Sher T., Hennessy B. T., Valero V. (2007). Primary angiosarcomas of the breast. *Cancer*.

[31] Sinnamon A. J., Neuwirth M. G., McMillan M. T. (2016). A prognostic model for resectable soft tissue and cutaneous angiosarcoma. *Journal of Surgical Oncology*.

[32] Wang L., Lao I. W., Yu L., Wang J. (2017). Clinicopathological features and prognostic factors in angiosarcoma: a retrospective analysis of 200 patients from a single Chinese medical institute. *Oncology letters*.

[33] Deyrup A. T., Weiss S. W. (2006). Grading of soft tissue sarcomas: the challenge of providing precise information in an imprecise world. *Histopathology*.

[34] Feinberg L., Srinivasan A., Singh J. K. (2018). Impact of specialist management on survival from radiation-associated angiosarcoma of the breast. *British Journal of Surgery*.

[35] Li G. Z., Fairweather M., Wang J., Orgill D. P., Bertagnolli M. M., Raut C. P. (2017). Cutaneous radiation-associated breast angiosarcoma. *Annals of Surgery*.

[36] Farzaliyev F., Hamacher R., Steinau Professor H. U., Bertram S., Podleska L. E. (2020). Secondary angiosarcoma: a fatal complication of chronic lymphedema. *Journal of Surgical Oncology*.

[37] Pasquali S., Pizzamiglio S., Touati N. (2019). The impact of chemotherapy on survival of patients with extremity and trunk wall soft tissue sarcoma: revisiting the results of the EORTC-STBSG 62931 randomised trial. *European Journal of Cancer*.

