# Supplementary material for: Localized Angiosarcoma, Not One Disease: A Retrospective Single-Center Study on Prognosis Depending on the Primary Site and Etiology
Source: Sarcoma. 2021 Sep 10;2021:9960085. doi: 10.1155/2021/9960085 (PMC8449723; doi:10.1155/2021/9960085)

**Supplementary Materials**

**Supplement Figure 1: Multivariate analysis**


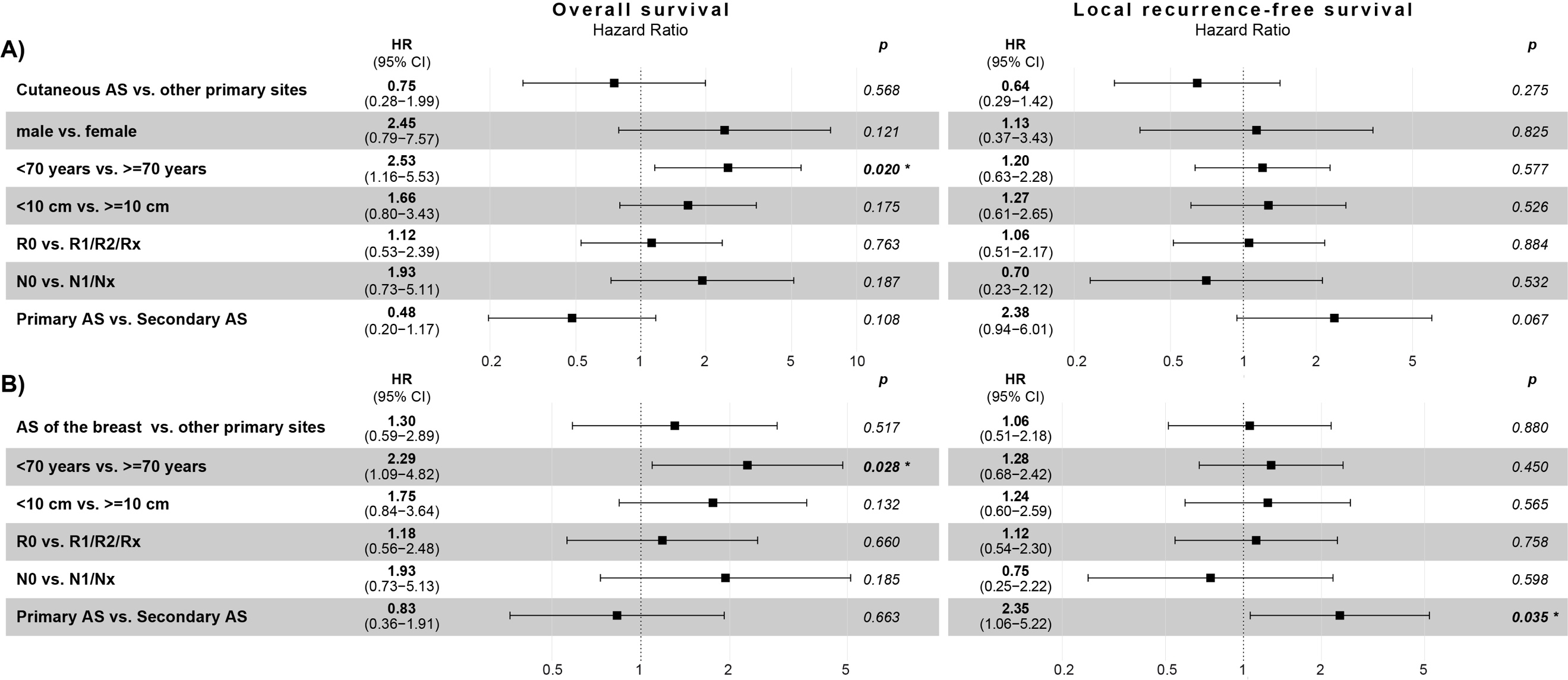


**Supplement Figure 2: Multivariate analysis metastasis-free survival**


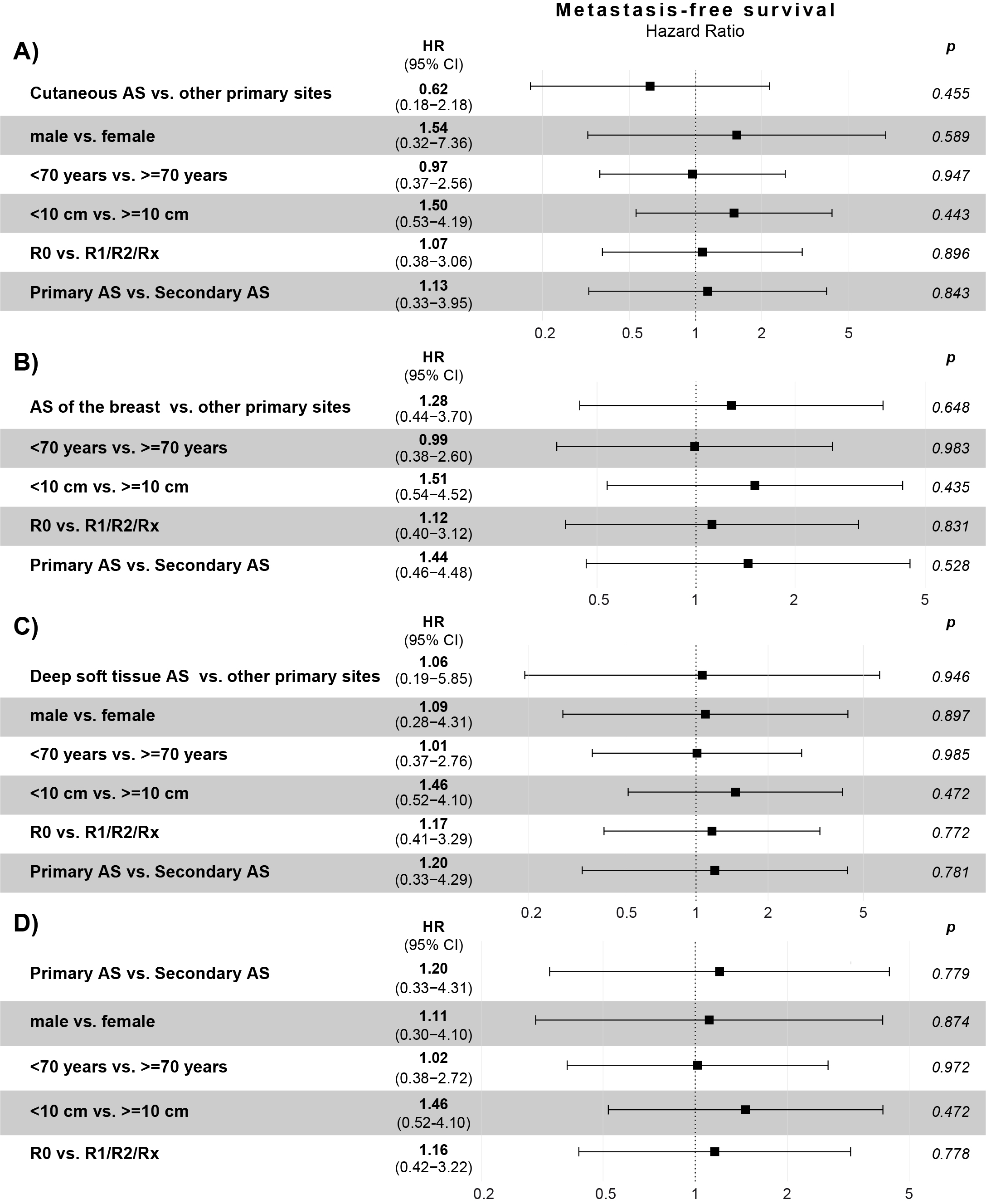

Supplement: Supplementary Materials — Supplement Figure 1: multivariate analysis. Forest plots of multivariate Cox regression analysis on overall survival (left) and local recurrence-free survival (right) for the subgroups A) cutaneous angiosarcomas and B) angiosarcomas of the breast showing hazard ratio (HR), 95% confidence interval (CI), and p value. Supplement Figure 2: multivariate analysis of metastasis-free survival. Forest plots of multivariate Cox regression analysis on metastasis-free survival for the subgroups A) cutaneous angiosarcomas, B) angiosarcomas of the breast, C) deep soft tissue angiosarcomas, and D) primary vs. secondary angiosarcomas showing hazard ratio (HR), 95% confidence interval (CI), and p value. [file 9960085.f1.docx]
